# Surgical versus trans-catheter aortic valve replacement (SAVR vs TAVR) in patients with aortic stenosis: Experience in a community hospital

**DOI:** 10.1097/MD.0000000000017915

**Published:** 2019-11-11

**Authors:** Tomer Ziv-Baran, Richard B. Zelman, Philip Dombrowski, Amber E. Schaub, Rephael Mohr, Dan Loberman

**Affiliations:** a Department of Epidemiology and Preventive Medicine, School of Public Health, Sackler Faculty of Medicine, Tel Aviv University, Tel Aviv, Israel; b Cape Cod Hospital, Hyannis, MA, USA; c School of Medicine, Sackler Faculty of Medicine, Tel Aviv University, Tel Aviv, Israel; d Division of Cardiac Surgery, BWH, Harvard Medical School, Boston, MA, USA.

**Keywords:** aortic valve replacement, community hospital, outcomes, SAVR, TAVR

## Abstract

Trans-catheter aortic valve replacement (TAVR) has become an alternative to surgical aortic valve replacement (SAVR) in high and intermediate risk patients with aortic stenosis. TAVR programs are spreading from large referral centers and being established in community based institutions. The purpose of this study was to compare the outcomes of TAVR to those of SAVR in a community hospital.

A historical cohort study of patients with aortic stenosis and pre-post procedure echocardiography data who underwent SAVR or TAVR in Cape Cod Hospital between January 2014 and December 2016. Patient characteristics and procedure outcomes were compared between the two procedures.

The study included 230 patients, of them 111 underwent SAVR and 119 underwent TAVR. None of the patients died during the 30 days after the procedure. TAVR patients had higher rates of postoperative mild+ aortic regurgitation (AR) (29.4% vs 12.6%, *P* = .002), postoperative atrial ventricular blocks (11.8% vs 0.9%, *P* = .001), and more often need an implantation of pacemaker (16.8% vs 0.9%, *P* < .001). Postoperative mean gradient of SAVR patients was higher (median 14 vs 11 mm Hg, *P* = .001) and atrial fibrillation postoperatively was more frequent (18.9% vs 2.5%, *P* < .001). Length of stay after procedure was shorter in TAVR patients (median 2 vs 4 days, *P* < .001).

After controlling for confounders, the use of TAVR was associated with an increased risk for postoperative pacemaker implantation (OR = 16.3, 95%CI 1.91–138.7, *P* = .011), lower mean gradient (−4.327, 95%CI −7.68 to −0.98, *P* = .011), and lower risk for atrial fibrillation (OR = 0.11, 95%CI 0.03–0.38, *P* = .001), but not with postoperative AR (OR = 0.84, 95%CI 0.22–3.13, *P* = .789).

In conclusion, short-term mortality was not reported in SAVR or TAVR patients. However, TAVR was associated with an increased risk for postoperative pacemaker implantation but with a lower risk for atrial fibrillation. Aortic valves implanted through a trans-catheter approach are also associated with a better hemodynamic performance.

## 1 Introduction

Trans-catheter aortic valve replacement (TAVR) was approved by the US Food and Drug Administration in late 2011 for the treatment of patients with severe asymptomatic aortic stenosis who are too ill or frail for the traditional surgical aortic valve replacement (SAVR).^[1,2]^ The procedure has rapidly gained the acceptance from patients and doctors , particularly for patients in their 80s and 90s due to the aging of the population.^[3–5]^ TAVR performed in experienced centers, with the use of a lower-profile, 2nd-generation device, was non-inferior to surgery with respect to death from any cause or disabling stroke at 2 years. Their bio-prosthetic-valve gradients were lower and the valve areas were greater, when compared with surgical valves.^[6]^ Similar findings were reported recently with the third generation TAVR prosthesis describing the outcome in intermediate risk aortic valve patients in recently published studies.^[7–9]^ A recent meta-analysis showed that TAVR may be an acceptable alternative to SAVR also in patients with intermediate risk for surgery.^[10]^ A study that summarized data from Virginia showed that implementation of TAVR was associated with the decrease in Society of Thoracic Surgeons (STS) risk of mortality and that the outcomes of SAVR continue to improve probably because of the availability of TAVR.^[3]^ The implantation of aortic trans-catheter valves (PATNER) trial reported 5-year outcomes of TAVR in high-risk patients and showed that it has similar outcomes when compared with SAVR.^[11]^ A study that examined the cost-effectiveness of TAVR versus SAVR from a US perspective revealed that the value of TAVR is higher than that of SAVR and an average of 4.4 days shorter length of stay.^[12]^ Excellent first year outcomes of TAVR in a low-volume center were reported in Canada.^[13]^ However, a comparison of outcomes in patients who underwent TAVR versus SVAR in a community center is still missing. Therefore, the purpose of this study was to compare the early outcomes of TAVR with those of SAVR performed in the same time period in a community hospital.

## 2 Methods

### 2.1 Study design and participants

This is a historical cohort study of patients who underwent SAVR or TAVR for aortic stenosis at Cape Cod Hospital (CCH) between January 2014 and December 2016. The TAVR program was established in CCH in June 2015. Patients who underwent SAVR between January 2014 and December 2016 and patients who underwent TAVR between June 2015 and December 2016 were compared. Patients without complete pre- and post-procedure trans-thoracic echo-cardiographs were excluded.

The study was approved by the Institutional Review Board of the Cape Cod Hospital. Informed consent was waived.

### 2.2 Setting

CCH is a 259-bed acute care community hospital located in Hyannis, MA with a 15-bed cardio-thoracic surgery department.

### 2.3 Variables and data source

Preoperative, operative, and postoperative data were obtained from review of medical records. Data collected included age, gender, prior percutaneous interventions (PCI), prior coronary artery bypass grafting (CABG), pre-procedure ejection fraction, mean trans-aortic gradient, calculated aortic valve area, aortic regurgitation (AR), mitral regurgitation, tricuspid regurgitation, and calculated pre-procedure STS probability of mortality (PROM). Intra-procedure data collected was the size of aortic valve prosthesis implanted. Postprocedural data on 30-day mortality, post-procedure strokes, arrhythmia, myocardial infraction (MI), and vascular complication and ECHO findings were collected using the CCH medical records data.

Baseline patient characteristics and in-hospital outcomes were collected according to The Society of Thoracic Surgeons Adult Cardiac Surgery Database Data Collection Form Version 2.81 (April 23 2015).^[14]^ A peri-operative MI was defined as the postoperative appearance of new Q waves or ST segment elevation of more than 2 mm on an electrocardiograph, accompanied by a creatinine phosphokinase-myocardial band greater than 50 mU/mL, with or without a regional wall motion abnormality.^[15]^ A cerebrovascular accident was defined as a new permanent neurological deficit and computed tomographic evidence of cerebral infarction.^[14,16]^

### 2.4 Bias

In order to avoid selection bias, all patients who underwent surgery during the study period were included in the study. We used a standard data collection form to avoid misclassification bias.

### 2.5 Study size

A significance level of 5% and a power of 80% were used to calculate the sample size.

One hundred twenty-eight and 88 patients were needed to identify a medium difference in continuous (effect size *d* = 0.5) and dichotomous (effect size *w* = 0.3) variables between the surgical methods, respectively.

### 2.6 Statistical analysis

Categorical variables were expressed as number and percentages. Distribution of continuous variables was assessed using histogram and Q–Q plots. Continuous variables were described as median and interquartile range (IQR). Categorical variables were compared using Chi-square test or Fisher's exact test and continuous variables were compared using independent samples *t* test or Mann–Whitney test.

In order to control the difference in baseline characteristics between the groups, a propensity score was calculated as the probability to have TAVR implantation. Logistic regression was used to calculate the propensity score using age, gender, aortic valve area, STS PROM, previous percutaneous intervention or coronary artery bypass grafting, ejection fraction, mean gradient, valve size, preoperative mitral regurgitation ≥ moderate, preoperative tricuspid regurgitation ≥ moderate, and preoperative AR ≥ mild. The propensity score was used for inverse probability of treatment (TAVR) weights (IPTW). Stabilized weights were calculated and univariate weighted logistic and linear regression models with robust standard errors were performed. A two-tailed *P* < .05 was considered statistically significant. Analyses were performed with SPSS (IBM Corp. Released 2016. IBM SPSS Statistics for Windows, Version 24.0. Armonk, NY: IBM Corp.).

## 3 Results

**Figure 1:**
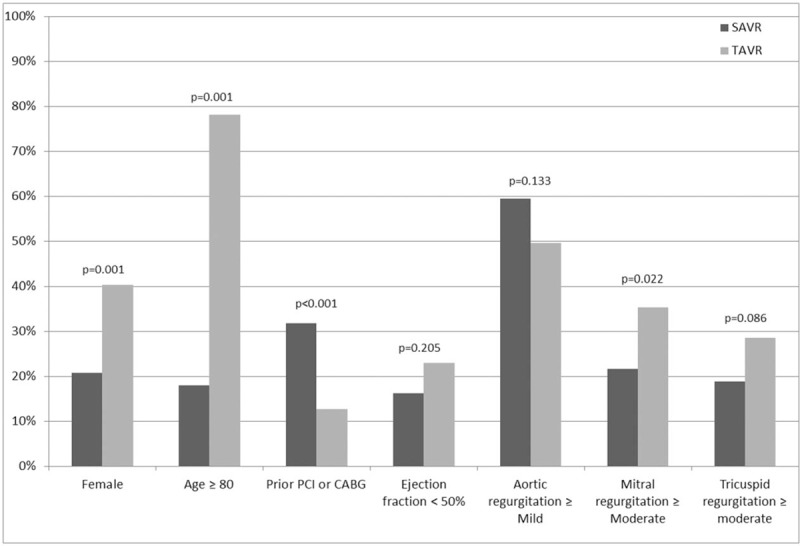
Comparison of baseline characteristics between patients who underwent TAVR and SAVR. SAVR = surgical aortic valve replacement, TAVR = trans-catheter aortic valve replacement.

**Figure 2:**
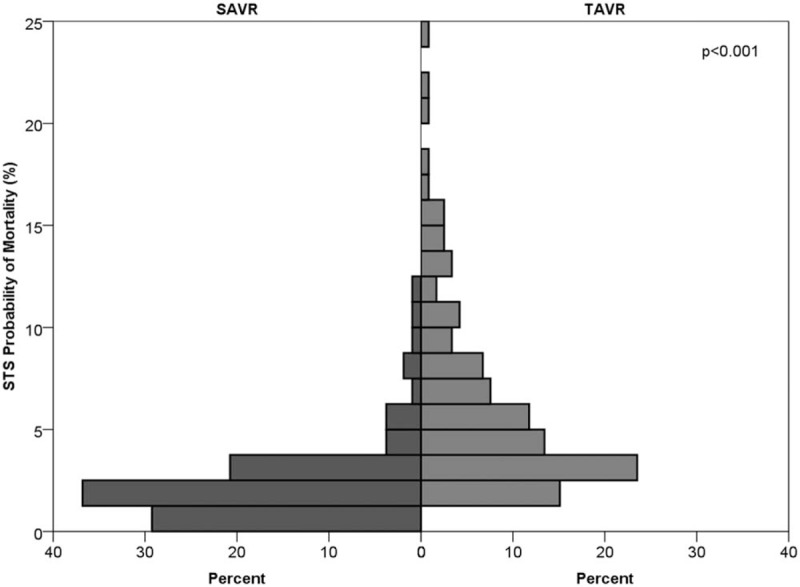
Mirror histogram presenting the STS probability of mortality in patients who underwent TAVR and SAVR. SAVR = surgical aortic valve replacement, STS = Society of Thoracic Surgeons, TAVR = trans-catheter aortic valve replacement.

Two hundred thirty patients were included in the study. Of them, 111 patients underwent SAVR and 119 underwent TAVR. Pre-procedure patient characteristics were significantly different between groups. Patients treated with TAVR were older (median 85, IQR 81–89 vs median 73, IQR 65–78, *P* < .001), more often female (40.3% vs 20.7%, *P* = .001), more likely to have moderate or severe MR (35.3% vs 21.6%, *P* = .022), had higher STS probability for mortality (median 4.8 vs 1.9, *P* < .001), smaller aortic valve area (median 0.7 vs 0.8, *P* = .026), and less likely to have prior PCI or CABG (12.6% vs 31.8%, *P* < .001). Comparison of preoperative patients’ characteristics is presented in Table 1 and Figures 1 and 2.

**Table 1:**
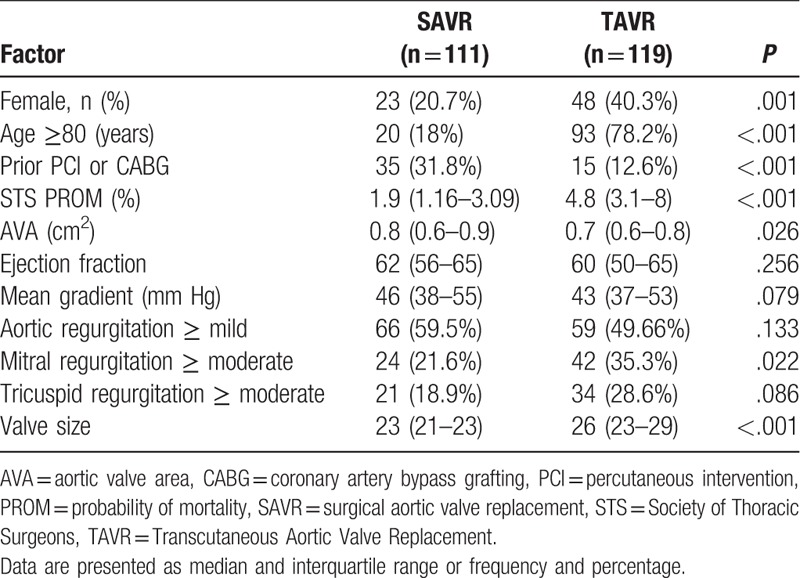
Preoperative characteristics and valve size in SAVR vs TAVR patients (N = 230).

The median implanted valve size in TAVR procedure (26 mm) was higher than the size used in SAVR procedure (23 mm, *P* < .001). Thirty days operative mortality was 0% in both groups. However, TAVR patients had higher rates of new postoperative mild+ AR (29.4% vs 12.6%, *P* = .002), postoperative atrial ventricular blocks (11.8% vs 0.9%, *P* = .001), and more often need an implantation of pacemaker (16.8% vs 0.9%, *P* < .001).

**Figure 3:**
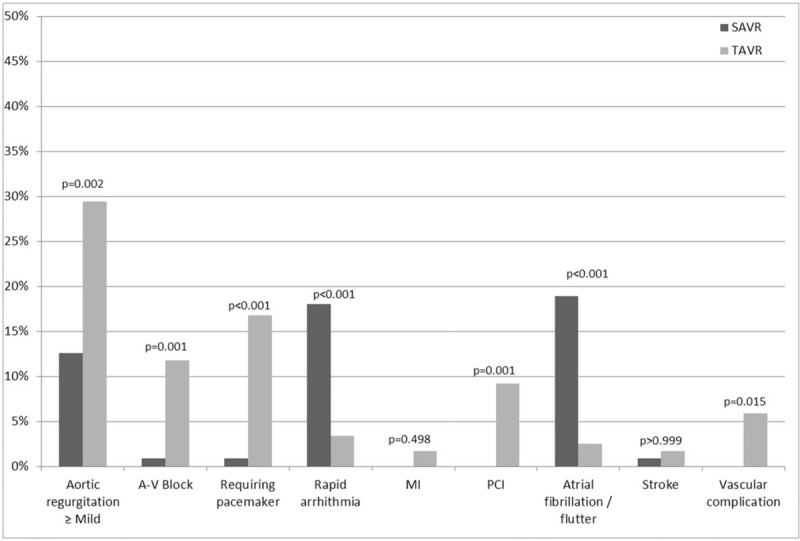
Comparison of outcomes between patients who underwent TAVR and SAVR. SAVR = surgical aortic valve replacement, TAVR = trans-catheter aortic valve replacement.

SAVR patients had more often rapid arrhythmia (18% vs 3.4%, *P* < .001), atrial fibrillation or flutter (18.9% vs 2.5%, *P* < .001), and had higher postoperative mean gradient (median 14 vs 11 mm Hg, *P* = .001). Median length of stay after the procedure was shorter in patients who underwent TAVR (2 vs 4 days, *P* < .001). Comparison of postoperative patients’ characteristics is presented in Table 2 and Figure 3.

**Table 2:**
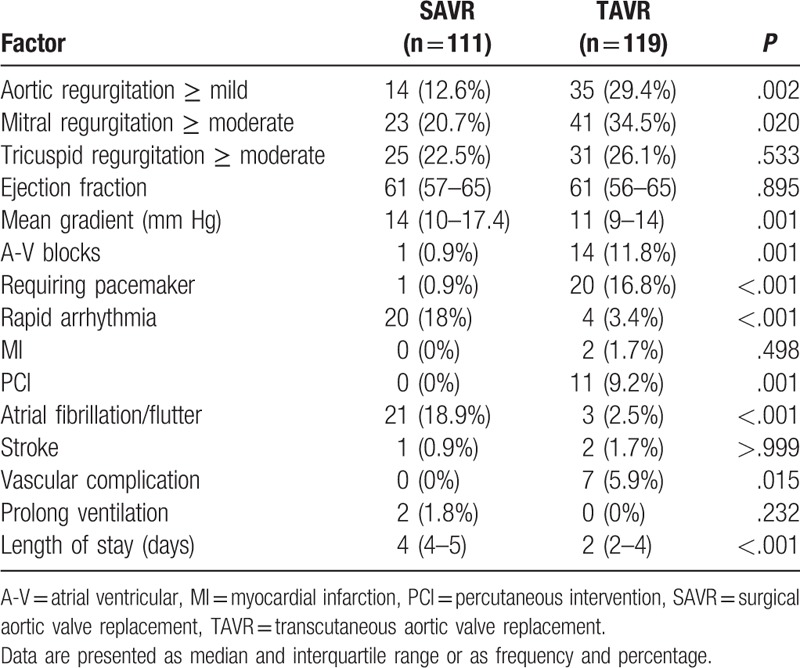
Postoperative status and complications in SAVR vs TAVR patients (N = 230).

After controlling the baseline characteristics using propensity score, TAVR patients had lower postoperative mean gradient (−4.327, 95%CI −7.68 to −0.98, *P* = .011) and were in an increased risk for a pacemaker implantation (OR = 16.3, 95%CI 1.91–138.7, *P* = .011). SAVR patients were in an increased risk for rapid arrhythmia (OR = 9.35, 95%CI 2.58–33.3, *P* = .001). There was no significant association between the type of procedure and postoperative AR (OR = 0.84, 95%CI 0.22–3.13, *P* = .789).

## 4 Discussion

SAVR reduces symptoms and improves survival in patients with aortic stenosis.^[1,6]^ Number of aortic valve procedures for the treatment of aortic valve stenosis has risen more than 60% since 2012. However, the increase in annual national totals is mainly related to more TAVR procedures performed.^[9]^ The number of SAVR procedures is expected to decrease significantly due to the popularization of the TAVR, which is a good and less invasive alternative.^[7–9]^ It has already been established that TAVR, performed in experienced centers, is non-inferior to surgery with respect to death or stroke at 5 years.^[14,11]^ However, the occurrences of postoperative AR and AV blocks are still higher than the average rates of these complications after SAVR.^[9]^ Gradually, in light of proven non-inferior results, and in light of aging population preferring medical care administered close to home and supportive environment, TAVR programs are being developed in community-based institutions. The TAVR program was established in CCH in June 2015. This report compares the outcomes of TAVR and SAVR in this community hospital.

The main findings in this report are the reduced rate of a postoperative pacemaker implantation associated with SAVR and the better hemodynamic performance of the TAVR prostheses reflected in their reduced postoperative mean gradient. TAVR patients also had shorter length of stay after the procedure as also reported previously.^[12]^

Aortic stenosis is the most common clinically significant form of valvular defect in adults.^[14]^ Treatment of aortic stenosis patients with TAVR has increased significantly the numbers of aortic stenosis patients amenable for treatment, who not long ago were regarded to be too sick or too frail to undergo SAVR.^[1]^ Further support for the expansion of the TAVR procedure was the development and popularization of techniques that enable its implantation in hybrid operating rooms or even catheterization laboratories without the use of extra-corporeal circulation.^[17]^

TAVR procedures at CCH have been performed mostly under local sedation, almost exclusively. This was possible mainly thanks to a very experienced team of anesthesiologists from a larger referral center, who work daily with our cardiac surgery team/catheterization lab, and an experienced interventional cardiologist. Hence, the relatively short period of learning curve.

The TAVR group in our study had bio-prosthetic-valve gradients lower than those of the SAVR group. The better hemodynamic performance of TAVR was reported in previous reports that showed lower gradients and greater valve areas in TAVR as compared with surgical valves.^[6]^ On the other hand, these studies as well as other reports have demonstrated significant number of patients with conduction disturbances and post-procedure AR similar to those demonstrated in our report.^[18,9]^

This study is a retrospective, and focuses on the comparison of early postoperative outcomes. Further investigation is required with larger number of patients and surgeons and longer follow-up.

In conclusion, TAVR is a procedure less invasive than SAVR with similar early outcome in a community hospital. Short-term mortality was not reported in SAVR or TAVR patients. However, TAVR was associated with increased risk for a postoperative pacemaker implantation but with lower risk for atrial fibrillation. Aortic valve prostheses implanted through TAVR approach are also associated with a better hemodynamic performance.

## Author contributions

**Conceptualization:** Dan Loberman, Philip Dombrowski, Rephael Mohr.

**Data curation:** Dan Loberman, Amber E. Schaub, Tomer Ziv-Baran.

**Formal analysis:** Rephael Mohr, Tomer Ziv-Baran.

**Investigation:** Dan Loberman, Rephael Mohr.

**Methodology:** Rephael Mohr, Tomer Ziv-Baran.

**Project administration:** Richard B. Zelman, Amber E. Schaub.

**Supervision:** Dan Loberman, Rephael Mohr.

**Validation:** Richard B. Zelman, Philip Dombrowski, Rephael Mohr.

**Writing – original draft:** Dan Loberman.

**Writing – review & editing:** Richard B. Zelman.

Dan Loberman orcid: 0000-0002-9634-1912.
